# Ophthalmologists beware of adenoviruses

**DOI:** 10.11604/pamj.2014.17.205.3153

**Published:** 2014-03-15

**Authors:** Hanan Handor, Rajae Daoudi

**Affiliations:** 1 Université Mohammed V Souissi, Service d'Ophtalmologie A de l'Hôpital des Spécialités, Centre Hospitalier Universitaire, Rabat, Maroc

**Keywords:** Ophthalmologists, adenoviruses, keratoconjunctivitis

## Image in medicine

Epidemic keratoconjunctivitis caused by different serotypes of human adenoviruses is an explosive and highly contagious ocular surface infection. This is the case of a young ophthalmologist who presented a filamentary keratitis complicating an adenoviral keratoconjunctivitis. Filamentary keratitis is a chronic and debilitating disorder related to dry eye syndrome. Patients with filamentary keratitis report ocular discomfort ranging from mild foreign-body sensation to severe ocular pain. Filaments are composed of mucin, and degenerating and regenerating epithelial cells of the cornea. Current management of filamentary keratitis involves treating the underlying dry eye (non preserved lubricants, topical steroidal agents, and punctal plugs) and mechanical removal of filaments.

**Figure 1:**
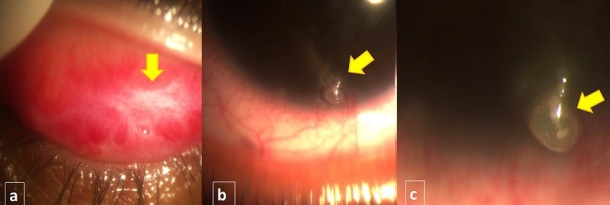
A) fibrosis of the tarsal conjunctiva in response to severe adenoviral Keratoconjunctivitis (yellow arrow); (B,C): mucus filament adhering to the corneal surface (yellow arrows)

